# Why study the use of animal products in traditional medicines?

**DOI:** 10.1186/1746-4269-1-5

**Published:** 2005-08-30

**Authors:** Rômulo RN Alves, Ierecê L Rosa

**Affiliations:** 1 Departamento de Biologia, Universidade Estadual da Paraíba and Programa de Pós-Graduação em Ciências Biológicas (Zoologia), Departamento de Sistemática e Ecologia, Universidade Federal da Paraíba, 58059-970 João Pessoa, PB, Brasil; 2 Departamento de Sistemática e Ecologia, CCEN, Universidade Federal da Paraíba, 58059-900 João Pessoa, PB, Brazil

## Abstract

The World Health Organization (WHO) estimates that as many as 80% of the world's more than six billion people rely primarily on animal and plant-based medicines. The healing of human ailments by using therapeutics based on medicines obtained from animals or ultimately derived from them is known as zootherapy. The phenomenon of zootherapy is marked both by a broad geographical distribution and very deep historical origins. Despite their importance, studies on the therapeutic use of animals and animal parts have been neglected, when compared to plants. This paper discusses some related aspects of the use of animals or parts thereof as medicines, and their implications for ecology, culture (the traditional knowledge), economy, and public health.

## Introduction

The World Health Organization (WHO) estimates that as many as 80% of the world's more than six billion people rely primarily on animal and plant-based medicines [1]. Traditional human populations have a broad natural pharmacopoeia consisting of wild plant and animal species. Ingredients sourced from wild plants and animals are not only used in traditional medicines, but are also increasingly valued as raw materials in the preparation of modern medicines and herbal preparations [2].

Animals and products derived from different organs of their bodies have constituted part of the inventory of medicinal substances used in various cultures since ancient times [3,6,7]; such uses still exist in traditional medicine. The healing of human ailments by using therapeutics based on medicines obtained from animals or ultimately derived from them is known as zootherapy [4]. As Marques [5] states, "all human culture which presents a structured medical system will utilize animals as medicines". The phenomenon of zootherapy is marked both by a broad geographical distribution and very deep historical origins.

In modern societies, zootherapy constitutes an important alternative among many other known therapies practiced worldwide. Wild and domestic animals and their by-products (*e.g*., hooves, skins, bones, feathers, tusks) form important ingredients in the preparation of curative, protective and preventive medicine [6,7]. For example, in Traditional Chinese Medicine (TCM), more than 1500 animal species have been recorded to be of some medicinal use [8]. In India nearly 15–20 percent of the Ayurvedic medicine is based on animal-derived substances [9]. In Bahia State, in the northeast of Brazil, over 180 medicinal animals have been recorded [10].

There are many reasons why studies on the use of animals, integrally or in parts, as medicines and their implications should be carried out and recorded. Among several approaches to be considered, this paper briefly discusses those concerning with the ecological, cultural (traditional knowledge), economical, and sanitary aspects of zootherapy.

## Ecological Approach

The world is facing potentially massive loss of wildlife due to over-hunting [11-13] and overfishing [14-17]. Transformation of ecosystems wrought through economic activities has been putting severe constraints on the availability and accessibility of specific types of plant and animal species used for medicinal purposes [18].

Regrettably, the demand created by traditional medicine is one of the causes of the overexploitation of the wild population of numerous animal species [19]. The use of animals in popular medicine certainly provokes pressure on natural resources exploited through traditional forms of collection, mainly due to general acceptance of popular medicine [20]. Medically speaking, the one major negative consequence of this trend is that there will be essentially less choice for the future development of medicines [19]. At present, about 40% of all prescription drugs are substances originally extracted from plants, animals, fungi and microorganisms [21].

Natural resource users may be the first to observe depletion [22]. However, as traditional peoples are integrated into the global economy, and come under trade, acculturation and population pressures, they lose their attachment to their own restricted resource catchments. This may lead to a loss of motivation in sustainable uses of a diversity of local resources, along with the pertinent indigenous knowledge [23].

Traditional ecological knowledge is of significance from a conservation perspective and an attribute of societies with continuity in resource use practice [23], therefore the dissociation of traditional knowledge from managerial ecology may result in the adoption of inadequate management options. Holders of traditional knowledge not only have a role as natural resource managers, but can also provide a model for biodiversity policies.

There is a need to shift the focus from how to obtain the greatest amount of zootherapeutical resources to how to ensure future uses. There is also a need for a transdisciplinary approach to integrate the various aspects of zootherapy in such a way that frameworks or methods to amalgamate ecological and social components of that practice can be increasingly tested. In this context, it is important not only to document the traditional uses of animal species, but also to integrate the cultural and biological aspects of such pratices into a broader discourse encompassing conservation, cooperative management, and sustainability.

## Cultural Approach

Human communities with historical practices of using resources acquire information of the ecosystem, process and local fauna and flora properties called Ecological Knowledge, which may be traditional, local or recently acquired [24-26]. Medicinal animals are important resources linking people to the environment and their use promotes the traditional lore related to them.

There is an increased interest in the knowledge that traditional populations possess on the use of animals for medicinal purposes, partly because the empirical basis developed throughout centuries may have, in many cases, scientific corroboration; but above all due to the historical, economical, sociological, anthropological, and environmental aspects of such a practice [3].

For centuries, healers and indigenous people have been collecting medicines from local plants and animals without threatening the population dynamics of the species because of the low level of harvesting. Loss of traditional knowledge has impact on the development of modern medicine. Medicinal folklore over the years has proved to be an invaluable guide in present day to the screening of important modern drugs (e. g., digitoxin, reserpine, tubocurarine, ephedrine, to name a few) that have been discovered by following leads from folk uses [18]. In view of this, it is evident the need to document the traditional knowledge of human communities, mainly because the majority of such communities are rapidly losing their socioeconomic and cultural characteristics.

The importance of protecting traditional knowledge and its cultural environmental resources is crucial, particularly in the context of globalisation and increased demand on natural resources worldwide. Traditional knowledge is valuable not only to those directly involved with it, but also to modern medicine and agriculture, among others. Moreover, protection of traditional knowledge can be used to raise the profile of the knowledge and its custodians. This not only has implications for the continuation of traditional practices within communities, but also for the interactions (e.g., economic, ecological) established outside the communities.

## Economical Approach

The value of biodiversity to human health has been highlighted in literature [27]. The most obvious benefit is the large proportion of the pharmaceutical armamentarium that is derived from the natural world. Over 50% of commercially available drugs are based on bioactive compounds extracted (or patterned) from non-human species [28]. Almost every class of drug includes a model structure derived from nature, exhibiting the classical effects of the specific pharmacological category. A great number of these natural products have come to us from the scientific study of remedies traditionally employed by various cultures [29]. In addition to plants and microbes, there has been increasing attention paid to animals, both vertebrates and invertebrates, as sources for new medicines. Animals have been methodically tested by pharmaceutical companies as sources of drugs for modern medical science [30], and the current percentage of animal sources for producing essential medicines is quite significant. Of the 252 essential chemicals that have been selected by the World Health Organization, 11.1% come from plants, and 8.7% from animals [31]. And of the 150 prescription drugs currently in use in the United States of America, 27 have animal origin [32].

Underlying the debate over traditional knowledge may be a much bigger issue such as the position of indigenous communities within the wider economy and society of the country in which they reside, and their access to or ownership of land they have traditionally inhabited. In that sense, concerns about the preservation of traditional knowledge, and the continued way of life of those holding such knowledge, may be symptomatic of the underlying problems that face these communities in the face of external pressures [33]

The trade in wildlife body parts and products includes traditional medicine, and it is well known that the annual global trade in animal-based medicinal products accounts for billions of dollars per year [31]. Nevertheless, in countries such as Brazil, the trade of animals for medicinal purposes has had little impact on the socioeconomic conditions of collectors, who generally are illiterate, underpaid, and perceive their activity as clandestine or semi-clandestine. The monetary value of animals sold for medicinal purposes in the country increases at each level of trade, and the socioeconomic profile of traders varies accordingly (I. L. Rosa and R. R. N. Alves, unpublished data).

Additionally, there is a need to assure that custodians of traditional knowledge receive fair compensation if the traditional knowledge leads to commercial gain, and to prevent appropriation of traditional knowledge by unauthorized parties [33].

## Sanitary Approach

Traditional drugs and traditional medicine in general represent a still poorly explored field of research in terms of therapeutic potential or clinical evaluation. There is a current preoccupation about this, since it is well-established that all sorts of vegetable, animal and mineral remedies used in a traditional setting are capable of producing serious adverse reactions. It is essential, however, that traditional drug therapies be submitted to an appropriate benefit/risk analysis [34]. Unfortunately, little research has been done so far to prove the claimed clinical efficacy of animal products for medicinal purposes [19].

Numerous infectious diseases can be transmitted from animals to humans (i.e. zoonoses). In this context, the possibility of transmitting infections or ailments from animal preparations to the patient should be seriously considered [19]. Several organs and tissues including bones and bile can be a source of *Salmonella *infection causing chronic diarrhoea and endotoxic shock. The possibility of transmission of other serious and widespread zoonoses such as tuberculosis or rabies should be considered whenever animal tissues from unknown sources are handled and used as remedies [35]. The possibility of toxic or allergic reactions to animal products should also be considered [36].

Broad categories of sanitary and phytosanitary regulatory measures are recognized for the food trade: 1) information measures which restrict the behaviour of suppliers only to the extent that they are required to disclose specified facts about their products; 2) measures that impose prior approval certifying that their products have met some pre-specified safety criteria before they can be released onto the market and 3) measures that allow suppliers to sell products without any prior official approval but imply that an offence is committed if the products fail to meet certain minimum safety standards [37]

The implementation of equivalent sanitary measures to the trade of animal or their parts for medicinal purposes poses considerable challenges, among them ensuring adequate participation of all stakeholders involved, combating illegal, unreported and unregulated trade, and monitoring of the activity.

## Final Considerations

Despite their importance, studies on the therapeutic uses of animals and their body parts have been neglected, when compared to plants [38]. Scholarly investigation of studies on medicinal uses of animals and their products, as well as of inorganic materials, should not be neglected and should be considered as an important complementary body of knowledge [3].

The extensive practice of traditional medicine in developing countries and the rapidly growing demand for alternative and basic therapeutic means (also in industrialized countries) constitute the international relevancy of research and development in the field of traditional drugs [39]. An additional motivation for such activities is found in the practical necessity to integrate the potential of traditional medicine into current practices of modern health care [39]. It is important to emphasize that some traditional medicinal systems, like the Chinese Traditional Medicine, is recognized by the WHO – World Health Organization [40] and accepted by one-fourth of the world human population.

There are numerous reasons to urgently re-think the medicinal use of animal products in traditional medicine both in humans and animals. In doing this, we should particularly take into account the rarity of some species, the unnecessary suffering involved in the harvesting (e.g., hunting, fishing) process, and the possible health risks linked to the administration of the animal-based remedies.

It is important to consider that human health is dependent on biodiversity and on the natural functioning of healthy ecosystems [41]. In this aspect, the use of animals for medicinal purposes is not simply a matter of the pharmaceutical and medical sciences; joint-research programmes should be undertaken with experts in the fields of ecology, linguistics, sociology, anthropology, etc. Thus, discussing zootherapy within the multidimensionality of sustainable development turns out to be one the key elements in achieving the sustenance of medicinal faunistic resources [10]. The use of endangered species in all forms of traditional medicine is a cause of growing concern.

Simultaneously, there is increasing dialogue between the conservation communities and traditional medicine communities globally. Showing respect and communicating in a language understood by all sides are not profound concepts. However, they demand time, money and goodwill [42]. Indigenous peoples have a storehouse of knowledge with regard to raw materials used in a range of products and processes, e.g., in agriculture, medicines, cosmetics, and foodstuffs, their knowledge of ecosystems being crucial to the care and management of biological diversity [43].

A growing respect for traditional knowledge has led modern science to adapt its procedures for assessing the impact of development projects on biological diversity; for monitoring of ecosystems, species, particular genetic resources and species at risk; for controlling alien species; and for promoting the in-situ conservation and sustainable management of biological diversity generally to identify but a few examples [44]. The use of animals for medicinal purposes is part of a body of traditional knowledge which is increasingly becoming more relevant to discussions on conservation biology, public health policies, sustainable management of natural resources, biological prospection, and patents.
